# Oral Erythromycin Improves the Quality of Endoscopy in Upper Gastrointestinal Bleeding Patients

**DOI:** 10.7759/cureus.10204

**Published:** 2020-09-02

**Authors:** Syed Asim Ali Shah, Muhammad Nadeem, Muzamil Jameel, Rifat Yasmin, Anum Afsar, Faiza Riaz

**Affiliations:** 1 Medicine, Wah Medical College, Wah Cantt, PAK; 2 Medicine, Poonch Medical College, Rawalakot, PAK

**Keywords:** erythromycin, endoscopy, placebo

## Abstract

Background

Upper gastrointestinal bleeding is a life-threatening emergency. Endoscopy is the therapeutic and diagnostic procedure of choice after initial stabilization of the patient. But the presence of retained blood, blood products, and other residual material in the stomach is a big challenge for endoscopists during urgent endoscopy after acute upper gastrointestinal bleeding. Intravenous erythromycin before endoscopy improves the visualization of gastric and duodenal mucosa in these patients. Use of oral erythromycin is more easy and convenient, so the objective of our study was to assess the effects of oral erythromycin on quality of endoscopy in upper gastrointestinal bleeding patients.

Methods

This interventional study was conducted at the Department of Medicine, POF Hospital Wah Cantt, Pakistan from January 2019 to December 2019. Patients with clinical evidence of acute upper gastrointestinal bleeding within 12 hours were inducted consecutively. Patients were randomly assigned to erythromycin (500 mg) suspension or placebo, orally three hours before endoscopy. One endoscopist performed all the procedures with the same double-channel video endoscope. The primary endpoint was endoscopic quality. The secondary endpoints were the need for second-look endoscopy within 48 hours, endoscopy related complications, therapeutic procedure performed or not during endoscopy, number of blood transfusions, and length of hospital stay.

Results

A total of 60 patients were included in the study; 30 received erythromycin and 30 received placebo. Out of these, 60% were male and 40% were female. The mean age was 53.68 ± 16.64. Quality of endoscopy was much better in the erythromycin group (83.3%) as compared to placebo (40%). Erythromycin did not shorten the endoscopic duration (15.53 vs. 14.33 minutes in the placebo group; p=0.216) and length of hospital stay (5.23 in erythromycin vs. 5.40 days in placebo group; p=0.807). Statistically no significant association was found between use of erythromycin and establishment of cause of bleed, need for second-look endoscopy, number of blood transfusions and number of endoscopic therapeutic procedures.

Conclusion

Erythromycin oral suspension before endoscopy in patients with acute upper gastrointestinal bleeding produced good quality of endoscopy in our study. It improved the visualization of gastric and duodenal mucosa significantly. However, it did not shorten the duration of endoscopy or hospital stay. There was no significant difference in number of second-look endoscopies and blood transfusions as well.

## Introduction

Upper gastrointestinal (GI) bleeding is a life threatening emergency and a common cause of hospital admission [1]. The incidence has decreased due to new treatment options especially for peptic ulcer disease but still it ranges from 46-67 cases per 100,000 [2,3]. Over the past two decades, new treatment modalities have been introduced for the management of upper gastrointestinal bleeding which have improved prognosis for these patients [4]. Despite this, upper GI bleeding still carries significant morbidity, mortality, and healthcare cost. In-hospital mortality is around 13% and chance of re-bleeding is 15% [5].

Endoscopy is the therapeutic and diagnostic procedure of choice after initial stabilization of patient in upper GI bleeding. Many endoscopic techniques are now used to treat this emergency [6,7]. Endoscopic treatment can improve immediate as well as delayed clinical outcomes. But the presence of retained blood, blood products, and other residual material in the stomach is a big challenge for endoscopists during urgent upper GI endoscopy after acute upper GI bleeding. This makes visualization difficult, leading to diagnostic and therapeutic problems during the procedure [8]. Different options like gastric lavage and instillation of 3% hydrogen peroxide were used to overcome this problem, results were good with hydrogen peroxide but sample size was very small, and results were not satisfactory with gastric lavage [9,10].

Researchers also tried erythromycin to improve the gastric motility and rapid clearance of blood and blood products from stomach to make endoscopy easy with promising results. Erythromycin used as antimicrobial agent is also motilin agonist and improves gastric emptying [11,12]. Many studies conducted in America and Europe showed that intravenous (IV) erythromycin 30 minutes before upper GI endoscopy in upper GI bleeding patients not only improves the visualization of gastric and duodenal mucosa also reduces the endoscopic duration and need of second-look endoscopy [13,14]. On the basis of these findings, the American Society for Gastrointestinal Endoscopy recommended the use of prokinetic drugs including erythromycin prior to endoscopy in patients with high probability of fresh blood or blood products in stomach [15]. The European Society of Gastrointestinal Endoscopy also recommends erythromycin administration prior to endoscopy in patients of upper GI bleeding [16].

Both of these societies recommend the use of IV erythromycin prior to endoscopy because all studies used IV erythromycin. Oral erythromycin is more easily available and also easy to administer. Oral erythromycin improves gastric emptying, acts rapidly, and can be used for rapid gastric emptying if IV form is not available [17]. With the same mechanism of rapid gastric emptying, oral erythromycin can improve the quality of endoscopy in upper GI bleeding. No work has been done with oral erythromycin in acute upper GI bleeding. In our region even no work has been done on IV erythromycin in acute upper GI bleeding to see its effect on the local population. Therefore, study of the role of erythromycin especially through oral route to see whether it improves the quality of endoscopy in our population was needed. The objective of this study was to assess the effect of oral erythromycin on the quality of the upper GI endoscopy in patients with acute upper gastrointestinal bleeding.

## Materials and methods

This interventional study was conducted at the Department of Medicine and Allied POF Hospital Wah Cantt from January 2019 to September 2109. Patients more than 18 years of age, either male or female, having history of hematemesis or melena in the last 12 hours irrespective of the cause of upper GI bleeding were included in the study consecutively. Patients having history of any kind of gastric or duodenal surgery, gastrokinetic drugs like metoclopramide, domperidone use in the last 24 hours, gastric lavage after hematemesis or melena, and known allergic to erythromycin were excluded. Pregnant women were also excluded. Informed consent was taken from the patient or a relative wherever relevant. Ethical approval was taken from Hospital Research Ethics Committee before start of the study.

All patients were admitted to the ICU and were managed accordingly and discharged when stable. Patients were kept nothing by mouth (NPO) at least eight hours before endoscopy. Endoscopy was performed within 12 hours of presentation in all patients. Patients were randomly assigned to erythromycin (500 mg) suspension or placebo orally given three hours before endoscopy, as peak levels are achieved after two hours of oral erythromycin [18]. Treatments were allocated using a computer randomization list at the hospital pharmacy. Only the pharmacist of the hospital delivered placebo or erythromycin suspension in identical packages. These packages were assigned numbers by the pharmacist and thus only he knew the result of randomization; neither the endoscopist nor patients were aware of the randomization. Treatment assignment was not released until the computer database is definitively frozen for final analysis.

One endoscopist performed all the procedures with the same double-channel video endoscope. The endoscopist performing the endoscopy on these patients was blinded to whether they were in the erythromycin or placebo group. Duration of endoscopy was recorded. Both primary and secondary endpoints were noted. The primary endpoint was endoscopic yield, as assessed by objective/subjective scoring system and endoscopic duration. This scoring system was taken from Carbonell et al. [19] and it was modified according to our needs. The subjective criteria were based on the endoscopist’s judgment. He noted whether stomach and duodenal mucosa were entirely visualized or not. He evaluated the quality of visualization by scoring (0- less than 25%, 1- 26% to 50%, 2- 51% to 75%, 3- more than 75% surface mucosa visible). The objective criteria were the presence or absence of clots in each area of stomach (fundus, corpus, antrum) and duodenum. Clots were strictly defined as clots that can’t be aspirated through accessory channel of the video endoscope. For each area (fundus, corpus, antrum, and duodenum) a score of 1 was considered when no clot was present and no score was considered on presence of clots. Based on subjective and objective criteria a total score of 5 or more out of 7 was considered as good quality and score below 5 was considered as poor quality of endoscopy. Whether diagnosis established or not was also noted during endoscopy. The secondary endpoints were the need for second-look endoscopy within 48 hours of first, endoscopic related complications, number of blood transfusions, and length of hospital stay. A standardized endoscopic report was established immediately after completion of endoscopy.

Data was entered in Statistical Product and Service Solutions (SPSS), version 20 (IBM Corp., Armonk, NY) and analyzed. A t-Test was used as t of significance for quantitative data and Chi-square was used to look for statistically significant association between different variables in both groups. A p-value <0.05 was considered as significant.

## Results

A total of 60 patients were included in the study; 30 received erythromycin and 30 received placebo. Out of these, 60% (n=36) were male and 40% (n=24) were female, mean age was 53.68 ± 16.64 ranging from 18 to 67 years, mean age for erythromycin group was 53.13±17.7 and for placebo group it was 54.23±15.8. Mean duration of endoscopy was 14.93±3.7 minutes, whereas mean hospital stay was 5.32±2.6 days. Variceal bleed was found in 43.3% (n=26), whereas 56.7% (n=34) had non-variceal bleed. Distribution in both groups is shown in Table 1.

**Table 1:** Distribution of variceal and non variceal bleeding in both groups

		Type of bleeding		Total
		Varceal bleed	Non Variceal bleed	
Drug used	Erythromycin	12	18	30
	Placebo	14	16	30
Total		26	34	60

Bleeding site was stomach in 53.3% (n=32), esophagus in 36.7% (n=22) and duodenum in 10% (n=6). Distribution in both groups shown in Table 2.

**Table 2:** Site of bleeding

		Site of bleed			Total
		Esophagus	Stomach	Duodenum	
Drug used	Erythromycin	11	17	2	30
	Placebo	11	15	4	30
Total		22	32	6	60

No significant side effects or endoscopic related complications were noted in either group. No death was recorded during the study period.

Oral erythromycin was associated with better endoscopic quality in erythromycin group (83%) vs in placebo group (40%) (better visibility and reduced number of blood clots) (Table 3).

**Table 3:** Endoscopic quality i.e visibility of mucosa and reduced number of blood clots

		Endoscopic quality		Total	p-value
		good	poor		
Drug used	Erythromycin	25	5	30	
	Placebo	12	18	30	.001
Total		37	23	60	

Erythromycin did not shorten the endoscopic duration and length of hospital stay; similarly there was no difference in number of blood transfusions (Table 4).

**Table 4:** Endoscopic duration, length of hospital stay and number of blood transfusions

											Drug used	N	Mean	Std. Deviation	Std. Error Mean	Sig. (2-tailed)
										Endoscopic duration in minutes	Erythromycin	30	15.53	4.289	.783	
											Placebo	30	14.33	3.032	.554	.216
										Length of hospital stay (in days)	Erythromycin	30	5.23	2.417	.441	
											Placebo	30	5.40	2.824	.516	.807
										Number of blood transfusions	Erythromycin	30	1.37	1.299	.237	
											Placebo	30	1.77	1.591	.290	.291

Statistically no significant association was found between use of erythromycin and establishment of cause of bleed and need for second-look endoscopy (Table 5).

**Table 5:** Need for second look endoscopy and diagnosis established during endoscopy

		Need for second look endoscopy			Total		p-value	
		yes	no					
Drug used	Erythromycin	4	26		30			
	Placebo	8	22		30		.333	
Total		12	48		60			
		Diagnosis established during endoscopy				Total		p-value
		yes		no				
Drug used	Erythromycin	30		0		30		
	Placebo	28		2		30		.492
Total		58		2		60		

## Discussion

Our results showed that oral erythromycin three hours before endoscopy was associated with better visibility at endoscopy, i.e. 83% good visibility in the erythromycin group vs 40% in the placebo group. There was no association between the use of erythromycin and establishment of cause of bleed and need for second-look endoscopy. Endoscopic duration and length of hospital stay was also not associated with use of erythromycin.

Many studies including randomized control trials have shown that use of erythromycin before endoscopy in upper GI bleeding helps in improving the quality of endoscopy [12,13,19]. A meta-analysis published in 2013 including seven randomized control trials showed that the administration of erythromycin before endoscopy in patients with upper GI bleeding significantly improves gastric mucosa visualization (odds ratio, 3.43; p<0.01) when compared with no erythromycin [20]. Another study conducted in Korea, which randomly assigned patients with upper GI bleeding to the erythromycin group without gastric lavage, gastric lavage only and erythromycin combined with gastric lavage concluded that satisfactory visualization of mucosa was achieved in more than 90% patients of the erythromycin group but only 60% in patients without erythromycin [21]. Our results are comparable with the above findings; we found that quality of endoscopy was good in 83% patients in the erythromycin group as compared to 40% in the placebo group with a p<.001. The European Society of Gastrointestinal Endoscopy already recommends the administration of erythromycin prior to endoscopy in non-variceal upper GI bleeding for better visualization [16]. All these prior trials used IV erythromycin; we could not find any data regarding the oral administration of erythromycin to compare our findings. Although studies have shown that oral erythromycin reduces gastric transit time significantly in capsule endoscopy supporting our findings [22,23].

Use of erythromycin did not change the duration of endoscopy or hospital stay in our study. Results in this regard are conflicting in previous trials; few studies have shown same results as in our study but most of the studies have opposite results. A study conducted by Carbonell et al. showed that erythromycin did not shorten the duration of endoscopy or duration of hospital stay, supporting our findings [19]. A study conducted in Switzerland concluded that erythromycin shortened the endoscopic duration, in contrast to our results, but did not affect the length of hospital stay comparable to our findings [24]. Two meta-analyses published in 2013 including many randomized control trials showed that use of erythromycin not only reduced the duration of endoscopy also reduced length of hospital stay, contrary to our findings [12,20]. 

Many studies have shown that use of erythromycin decreases the need for second-look endoscopy. Studies conducted by Coffin et al. and Fossard el al. showed that erythromycin significantly reduced the need for second-look endoscopy in patients with upper GI bleeding [24,25]. The same was concluded in a meta-analysis published in 2016 [14]. In our study, although only four patients underwent second-look endoscopy as compared to eight patients in the placebo group, the statistical difference was not significant (p=0.33) contrary to findings discussed earlier. This difference may be due to different route of administration and sample size.

Endoscopic related complications, number of blood transfusions and therapeutic procedures during endoscopy did not differ significantly among erythromycin and placebo group. Similar findings were observed by Na et al. and Carbonell et al. [19,21].

Our study had some limitations, first as it was a single-center study having 60 patients. Secondly all patients of upper gastrointestinal bleeding regardless of cause (variceal or non-variceal bleed) were included in the study. Multicenter studies with sufficient sample size comparing both intravenous and oral erythromycin suspension with placebo separate in variceal and non-variceal bleed are recommended. This study however opens a new horizon for oral use of erythromycin before upper GI endoscopy, which is the most important finding.

## Conclusions

Oral erythromycin suspension improved the quality of upper GI endoscopy in patients with acute upper gastrointestinal bleeding. It improved the visualization of gastric and duodenal mucosa significantly similar to other international studies with intravenous erythromycin, but it did not shorten the duration of endoscopy or hospital stay. There was no significant difference in number of second-look endoscopies and blood transfusions as well.

Oral erythromycin can be used to improve the quality of upper gastrointestinal endoscopy in patients with high probability of fresh blood or blood products in stomach, if intravenous erythromycin cannot be used due to any reason.
